# The paternal influence on early childhood development in Africa: implications for child and adolescent mental health

**DOI:** 10.1186/s13034-024-00847-4

**Published:** 2024-12-06

**Authors:** Augustus Osborne, Bright Opoku Ahinkorah

**Affiliations:** 1 Department of Biological Sciences, School of Basic Sciences, Njala University, PMB, Freetown, Sierra Leone; 2 REMS Consultancy Services, Takoradi, Sekondi-Takoradi Ghana; 3 Faculty of Health and Medical Sciences, The University of Adelaide, Adelaide, Australia

**Keywords:** Paternal influence, Child development, Father involvement, Early childhood

## Abstract

This commentary examines the influence of fathers in early childhood development in Africa and its implications for child and adolescent mental health. Historically overshadowed by maternal influence, research increasingly highlights the multifaceted impact of paternal involvement on children’s cognitive, emotional, social, and behavioural development. Fathers contribute uniquely to children’s mental growth through stimulating play and rich language interaction. Their emotional engagement fosters children’s self-esteem and resilience. Moreover, fathers influence social skills by modelling interactions and encouraging exploration. Paternal involvement is linked to improved behaviour regulation. Beyond direct interaction, factors such as paternal mental health, economic stability, and co-parenting dynamics also shape child outcomes. Despite the impact of engaged fatherhood on children’s cognitive, emotional, and social development, many fathers encounter barriers such as economic pressures, cultural norms, and migration. These challenges often hinder their ability to participate actively in their children’s lives, resulting in a disconnect that can affect family dynamics and child well-being. Addressing cultural and societal barriers to father involvement is crucial to optimise child development. To address these issues, the paper outlines several key policy implications aimed at promoting paternal involvement. This commentary serves as a foundation for further exploration of fathers’ complex and vital role in shaping children's lives.

## Background

The role of fathers in early childhood development is a crucial yet often overlooked aspect of childrearing, particularly in the African context [1]. Traditionally, discussions surrounding child development have focused predominantly on maternal influences, with less emphasis placed on the significant impact that fathers can have on their children’s growth and well-being [2]. However, research highlights the importance of paternal involvement, which encompasses a range of behaviours and interactions, including emotional support, financial provision, and active engagement in a child’s life [3].

Early childhood development is a critical period that shapes cognitive, social, and emotional growth, laying the foundation for a child’s future well-being and success [4].During these formative years, children are susceptible to their environment and the relationships they form. The presence and involvement of a father can be pivotal in fostering a secure attachment, encouraging exploration, and promoting resilience [5]. Research has shown that father involvement is linked not only to cognitive and emotional outcomes but also to a range of behavioral outcomes, including attachment security [6] and the development of internalizing [7] and externalizing behavior problems [8]. In many African cultures, the role of fathers is often defined by traditional norms and expectations, which can vary significantly across different communities and socio-economic contexts [9]. Understanding these dynamics is essential, as they can influence both the quality of father-child interactions and the subsequent developmental outcomes for children.Understanding paternal influence in Africa is especially important given the continent’s setting. In some societies, fathers are seen primarily as providers; in others, they are expected to take a more active role in nurturing and educating their children [10]. This variation can affect how children perceive their fathers and the types of relationships they develop. Moreover, socio-economic factors, such as poverty, migration, and urbanisation, further complicate the dynamics of fatherhood in Africa, often leading to challenges that can hinder a father’s ability to engage fully in his child’s life [11]. It is crucial to delineate the specific predictive power of paternal involvement in addition to maternal caregiving. Recent studies indicate that while both maternal and paternal involvement are vital, the unique contributions of fathers can lead to distinct outcomes in areas such as emotional regulation, social competence, and academic achievement [12]. However, the assertion that any paternal involvement positively affects child outcomes requires further investigation. Evidence suggests that the quality of paternal investment may be more critical than the quantity. High-quality engagement—characterized by emotional support, responsiveness, and active participation—has been shown to associate more strongly with positive child outcomes than mere presence or frequency of interaction [13]. Thus, it is essential to consider not only how often fathers are involved but also how they engage with their children.

It is essential to clarify the roles of caregivers in child development. Mothers are typically recognized as the primary caregivers, providing foundational emotional support and nurturing [14]. However, fathers also play a significant role in a child’s life, contributing uniquely to their development. Research indicates that paternal involvement can lead to distinct positive outcomes, particularly in areas such as emotional regulation, social competence, and academic achievement [3]. While the commentary refers to the “unique parenting style” of fathers in contrast to maternal figures, it is important to emphasize that the roles of secondary caregivers—whether they are fathers, grandparents, or other caregivers—are not interchangeable. Each caregiver brings different strengths and perspectives to the child’s upbringing. The presence of a male caregiver can offer unique benefits, but it is the quality of engagement, rather than the gender of the caregiver alone, that significantly influences child outcomes [2].This commentary aims to explore the multifaceted impact of fathers on early childhood development in Africa, highlighting both the positive contributions they can make and the challenges they face. By examining the various dimensions of paternal involvement, and the outcomes associated with this involvement, we can gain a deeper understanding of the pivotal role fathers play in shaping their children’s future. Ultimately, recognising and enhancing paternal roles is essential for fostering healthier developmental outcomes in children across the continent, paving the way for a more supportive and nurturing environment for future generations.

## Main text

### Paternal involvement in early childhood

Paternal involvement in early childhood encompasses a variety of roles and activities that fathers engage in to support their children’s development [3]. This involvement is crucial, as it influences children’s cognitive, emotional, and social outcomes. Understanding the different dimensions of paternal involvement and the contextual factors that shape these roles provides insight into how fathers can effectively contribute to their children’s growth.

In recent years, there has been a notable shift in fatherhood roles globally. For instance, research indicates that fathers in Australia, Denmark, France, Italy, and the United States showed a three- to six-fold increase in hours spent on childcare compared to what their own fathers typically did [15]. This evolution reflects changing societal norms around fatherhood and the increasing recognition of the importance of paternal involvement in child development. However, much less is known about similar changes in non-Western and less developed countries, including those in Africa. The dynamics of fatherhood in these contexts are influenced by various factors, including cultural expectations, economic conditions, and historical legacies [10]. Understanding these influences is crucial for a comprehensive view of paternal involvement. In African societies, the roles of fathers can vary significantly. In some cultures, fathers are primarily viewed as providers, while in others, they take on more nurturing roles [14]. This variation affects how children perceive their fathers and the relationships they develop. While mothers are typically recognized as the primary caregivers [14], fathers also contribute uniquely to child development, with their involvement linked to positive outcomes in emotional regulation, social competence, and academic success [12]. It is essential to consider both historical and cross-cultural perspectives when examining paternal involvement. The increasing engagement of fathers in various parts of the world highlights a shift toward recognizing the importance of their role.

### Key dimensions of paternal-involvement 

**Emotional Support:** Emotional support from fathers includes behaviours such as affection, encouragement, and active listening [16]. Fathers who provide emotional support help foster a sense of security and self-worth in their children [17]. Research indicates that children with emotionally supportive fathers tend to exhibit higher levels of self-esteem and better emotional regulation [18]. In African contexts, where traditional gender roles may discourage emotional expression, promoting emotional involvement can be transformative for both fathers and children.

**Financial Provision**: Traditionally, fathers have been viewed primarily as breadwinners, responsible for the family’s financial well-being [19]. In many African societies, this role is critical, as economic stability directly affects children’s access to education, healthcare, and nutrition [20]. However, while financial provision is essential, it is equally important for fathers to balance this with emotional and practical involvement in their children’s lives. The shift towards recognising the importance of holistic fatherhood can enhance the overall well-being of families.

**Educational Engagement:** Educational involvement involves fathers participating in their children’s formal and informal learning processes [21]. This includes helping with homework, attending school events, and fostering a learning environment at home. Studies show that children whose fathers are actively involved in their education tend to perform better academically and develop a love for learning [22]. In Africa, where educational disparities exist, fathers can play a pivotal role in advocating for their children’s educational needs and aspirations.

**Physical Play and Activities:** Engaging in physical play is another vital aspect of paternal involvement. Fathers often encourage active play, essential for developing motor skills, physical health, and social interactions [16]. Activities such as sports, outdoor play, and creative games promote physical development and strengthen the father-child bond [23]. In many African communities, traditional games and cultural practices can be integrated into play, enriching the developmental experience for children.

### Contextual factors influencing paternal involvement

The level and nature of paternal involvement can vary based on several contextual factors:

**Urban vs. Rural Settings:** In urban areas, fathers may have more access to resources, educational opportunities, and community programs that encourage involvement [24]. Conversely, rural fathers may face economic challenges and limited access to facilities, impacting their ability to engage fully with their children. However, rural fathers often have more informal learning and bonding opportunities through agricultural activities and community gatherings [25].

**Cultural Norms and Expectations:** Cultural beliefs about masculinity and fatherhood significantly shape paternal involvement. In some African cultures, traditional views may dictate that fathers focus primarily on financial provision, limiting their engagement in caregiving activities [26]. However, there is a growing recognition of the importance of nurturing roles, leading to shifts in cultural narratives that promote more balanced parenting [27].

**Economic Status:** Economic factors can significantly influence paternal involvement. Fathers facing financial stress may prioritise work over spending time with their children, impacting their ability to engage in emotional and educational support [28]. Conversely, fathers in more stable economic situations may have the resources and time to invest actively in their children’s development.

**Migration and Absenteeism:** In many African countries, migration for work is common, leading to absentee fathers [29]. This phenomenon can create gaps in paternal involvement, affecting children’s emotional and social development. Programs that support fathers in maintaining connections with their children, even from a distance, can mitigate some of the negative impacts of absenteeism.

### Impact of paternal influence on child mental health

The influence of fathers on their children’s development is profound and multifaceted, impacting various aspects of growth, including cognitive, emotional, social, and physical development [30]. Research has increasingly highlighted the critical role that paternal involvement plays in shaping these outcomes, particularly during early childhood, a formative period that lays the groundwork for lifelong skills and behaviours [3].

#### Cognitive development

Cognitive development refers to the progression of thinking, problem-solving, and learning abilities in children [31]. Fathers who actively converse with their children contribute to better language acquisition and vocabulary development. Studies have shown that children whose fathers frequently read to them or engage in storytelling demonstrate higher language skills than their peers [32]. This verbal interaction stimulates cognitive processes and encourages curiosity and exploration. Research indicates that children with involved fathers perform better academically [33]. Fathers who participate in school activities, help with homework and instil a love for learning positively influence their children’s educational outcomes. For instance, a study in South Africa found that children with engaged fathers were more likely to achieve higher grades and show increased motivation in school [34]. Fathers often engage in activities that promote critical thinking and problem-solving. Playful interactions, such as building blocks or strategic games, encourage children to think creatively and develop logical reasoning skills. This hands-on engagement fosters cognitive flexibility and resilience, equipping children with essential skills for future challenges.

#### Emotional development

Emotional development encompasses understanding, expressing, and regulating emotions [35]. Paternal involvement plays a significant role in shaping emotional well-being. Secure attachment to fathers fosters emotional stability and resilience [36]. Children who experience positive interactions with their fathers are more likely to develop secure attachments, leading to healthier emotional regulation and coping mechanisms. This emotional foundation is crucial for navigating social relationships and handling life’s challenges. Fathers who provide emotional support and encouragement help build their children’s self-esteem. Positive reinforcement from fathers can enhance children’s sense of self-worth, leading to confidence in their abilities. In African contexts, where traditional norms sometimes limit emotional expression, fostering open communication can significantly impact a child’s emotional health. Engaged fathers model emotional expression and empathy, teaching children how to navigate their feelings and understand others’ emotions [37]. This modelling is essential for developing social skills as children learn to communicate effectively, resolve conflicts, and build meaningful relationships.

#### Social development

Social development involves the acquisition of social skills and the ability to interact with peers and adults [38]. Paternal influence is crucial in shaping children’s social behaviours. Fathers who encourage social interactions and playdates help children develop essential social skills. Group activities teach children how to cooperate, share, and negotiate, which are vital for forming healthy peer relationships. Research has shown that children with involved fathers are likelier to have positive friendships and exhibit prosocial behaviours [39]. Active paternal involvement is associated with better behavioural regulation in children [39]. Fathers can play a pivotal role in teaching children about cultural values and social norms in diverse African societies. By engaging in community activities and cultural practices, fathers help instil a sense of identity and belonging, which is crucial for social integration and understanding one’s place in the community.

#### Physical development

Physical development refers to the growth and maturation of the body and motor skills [40]. Paternal involvement significantly contributes to this aspect of development. Fathers who often engage in physical activities with their children, promote exercise and physical health in children. Playful interactions, such as sports, outdoor games, and active play, enhance motor skills and coordination. Research indicates that children with active fathers are more likely to maintain a healthy weight and develop lifelong physical activity habits [41]. Fathers’ involvement in their children’s healthcare decisions improves health outcomes [42]. This includes promoting healthy eating habits, ensuring regular check-ups, and addressing health concerns promptly. In many African contexts, where access to healthcare may be limited, paternal involvement can significantly impact children’s overall health and well-being.

### Challenges faced by fathers in Africa

Fathers in Africa encounter various challenges that can significantly impact their ability to effectively engage in their children’s lives [43]. These challenges are multifaceted, stemming from socio-economic, cultural, and structural factors [43]. Understanding these obstacles is crucial for developing targeted interventions and support systems that enable fathers to fulfil their roles in child development more effectively.

#### Economic pressures

One of the most significant challenges for fathers in Africa is economic pressure [44]. Many fathers are expected to be the primary breadwinners, which can be particularly daunting due to high unemployment rates among youth and unskilled labourers [26]. This economic instability can limit fathers’ financial ability to provide for their families, leading to stress and feelings of inadequacy [45]. A substantial portion of the workforce in Africa is engaged in informal employment, which often lacks job security, benefits, and consistent income [46]. This precarious economic situation can hinder fathers’ ability to invest time and resources in their children’s development. High poverty levels can restrict access to essential resources such as education, healthcare, and nutrition [45]. Fathers struggling to meet basic needs may prioritise work overactive involvement in their children’s lives, leading to a disconnect between fathers and their families [47].

#### Cultural norms and expectations

Cultural beliefs and societal norms surrounding masculinity and fatherhood can create additional challenges for fathers [48]. In many African cultures, traditional gender roles dictate that fathers should primarily focus on financial provision, often sidelining their nurturing and emotional responsibilities [49]. This narrow definition of fatherhood can limit fathers’ involvement in caregiving and emotional support. Cultural norms discouraging emotional expression can hinder fathers from forming close emotional bonds with their children [3]. Fathers may feel pressured to conform to stoic ideals, preventing them from engaging in nurturing behaviours vital for healthy child development [50]. Many young men grow up without positive male role models in communities where father absenteeism is common [51]. This lack of guidance can perpetuate cycles of disengagement and limit the understanding of effective fatherhood.

#### Migration and absenteeism

Migration for work is a prevalent phenomenon in many African countries, leading to significant challenges for fathers [52]. Many fathers migrate to urban areas or other developed countries for better economic opportunities, often leaving their families behind [53]. This separation can create emotional distance and hinder the development of solid father-child relationships. Even when fathers are separated from their families, maintaining communication can be challenging due to technological barriers, financial constraints, or cultural differences [54]. This lack of interaction can exacerbate disconnection and impact children’s emotional well-being. Fathers who return after extended absences may struggle to reintegrate into family life [55]. Children may have developed different attachment styles or coping mechanisms during their absence, leading to potential conflicts and misunderstandings.

#### Lack of support systems

Fathers in Africa often lack institutional and community support, which can impede their ability to engage effectively in their children’s lives [1]. Many communities lack access to parenting education and support programs that can help fathers develop skills for effective involvement [43]. Without guidance, fathers may feel uncertain about their roles and responsibilities. Access to resources such as healthcare, childcare, and educational support can be limited, particularly in rural areas [56]. This lack of resources can hinder fathers’ ability to provide for their children’s physical and emotional needs. In some communities, fathers who take on non-traditional roles (e.g., stay-at-home dads or those who prioritise caregiving) may face stigma or judgment [19]. This societal pressure can discourage fathers from engaging in nurturing behaviours and seeking help when needed.

#### Health issues

Health challenges can also significantly impact fathers’ ability to participate in their children’s lives [57]. Many fathers can face health issues that can limit their physical capabilities or economic productivity [57]. Chronic illnesses, disabilities, and mental health challenges can create barriers to active participation in parenting [58]. In some communities, substance abuse can be a significant issue that affects fathers’ ability to engage positively with their children [59]. Substance dependency can lead to neglect, emotional unavailability, and strained family relationships [60]. Mental health issues are often stigmatised in many African cultures, preventing fathers from seeking help or support [61]. This stigma can exacerbate feelings of isolation and inadequacy, further hindering their involvement in family life.

#### Educational barriers

Educational challenges can also affect fathers’ engagement in their children’s development [62]. Many fathers may have limited educational backgrounds, affecting their confidence in supporting their children’s learning and development [63]. This lack of educational attainment can hinder their ability to engage in academic activities with their children. Fathers may lack access to information about child development, effective parenting strategies, and available resources [43]. This knowledge gap can limit their ability to provide the necessary support for their children’s growth.

### Policy implications for promoting paternal involvement in Africa

Promoting paternal involvement in child development is essential for fostering healthier families and communities across Africa. Policymakers play a crucial role in creating an environment that supports fathers in their caregiving roles.

#### Family-friendly workplace policies

Governments should advocate for and implement policies that provide adequate paternity leave, allowing fathers to take time off work to bond with their newborns. This leave should be paid and long enough to encourage active involvement in early caregiving. They should also encourage employers to adopt flexible work arrangements, such as remote work or adjusted hours, to help fathers balance work and family responsibilities. Policies encouraging companies to create family-friendly environments can lead to more engaged fatherhood.

#### Parenting Education and Support Programs

Governments and NGOs should invest in parenting education programs that specifically target fathers. These programs can cover child development, effective communication, and emotional support, equipping fathers with the skills necessary for active involvement. They should also create and fund community centres that offer support groups, workshops, and resources for fathers. These centres can serve as hubs for fathers to connect, share experiences, and receive guidance on parenting challenges.

#### Public Awareness campaigns

Governments should initiate public awareness campaigns highlighting the importance of father involvement in child development. These campaigns can feature testimonials from engaged fathers and promote positive fatherhood narratives that challenge traditional stereotypes. Leverage social media, radio, and television to disseminate information about the benefits of paternal involvement could also be useful. Collaborating with local influencers and community leaders can enhance the reach and impact of these campaigns.

#### Legal frameworks and child support

Governments should strengthen legal frameworks that ensure fathers fulfil their financial responsibilities while promoting emotional and practical involvement in their children’s lives. Implementing fair child support systems can alleviate economic pressures on fathers, allowing them to engage more fully. Developing legal protections that recognise fathers’ rights to participate in decision-making regarding their children’s education, healthcare, and upbringing is worthwhile. This can ensure that fathers have a voice in these areas can enhance their involvement.

#### Health and social services integration

Governments should create programs that integrate health, education, and social services to provide comprehensive support for families. Policymakers can facilitate paternal involvement by addressing the various needs of families, including economic, health, and educational support. There should also be increase in access to mental health services for fathers, recognising that emotional well-being is crucial for effective parenting. Providing counselling and support for issues such as stress, depression, or substance abuse can help fathers engage more positively with their children.

## Conclusion

Promoting paternal involvement in Africa is not merely a matter of enhancing individual family dynamics; it is a collective investment in the future of societies. By recognising fathers’ critical role in child development and addressing the barriers they face, we can contribute to creating healthier, more resilient generations. As we look to the future, fostering an environment where fathers feel empowered and supported in their roles is essential. This vision requires a commitment from all sectors of society to prioritise fatherhood, challenge stereotypes, and create policies that enable fathers to engage meaningfully in their children’s lives. Ultimately, the journey toward promoting positive paternal involvement is a shared responsibility that holds the promise of transforming families and communities across Africa. By valuing and supporting fathers, we can pave the way for a brighter, more equitable future for all children.

The involvement of fathers in child development has profound implications for the mental health and well-being of children and adolescents. Fathers play a crucial role in providing emotional security and attachment, which are essential for healthy mental development in early childhood. Active paternal engagement fosters better psychological well-being, reduces anxiety, and supports cognitive development, helping children manage stress and challenges more effectively. Additionally, children with engaged fathers tend to exhibit fewer behavioral problems, reducing the risk of conditions such as attention-deficit/hyperactivity disorder and conduct disorders. Fathers who are emotionally present contribute significantly to the development of social skills, emotional intelligence, and a positive self-concept in children, laying the foundation for healthier mental and emotional resilience.

 When barriers prevent fathers from being fully engaged, these emotional and psychological benefits are diminished, which can lead to long-term mental health challenges. Prioritizing paternal involvement can therefore play a key role in ensuring the mental well-being of future generations across Africa.

## Data Availability

No datasets were generated or analysed during the current study.
